# Enduring voice recognition in bonobos

**DOI:** 10.1038/srep22046

**Published:** 2016-02-25

**Authors:** Sumir Keenan, Nicolas Mathevon, Jeroen MG Stevens, Jean Pascal Guéry, Klaus Zuberbühler, Florence Levréro

**Affiliations:** 1 Université de Lyon/Saint-Etienne, Equipe Neuro-Ethologie Sensorielle, ENES/Neuro-PSI, CNRS UMR 9197, Saint-Etienne, France; 2 University of St. Andrews, School of Psychology & Neuroscience, St. Andrews, Scotland, UK; 3 Department of Psychology, Hunter College, CUNY, New York, USA; 4 Royal Zoological Society of Antwerp, Centre for Research and Conservation, Antwerp, Belgium; 5 Vallée des Singes Zoological Park, Romagne, France; 6 Université de Neuchâtel, Department of Comparative Cognition, Neuchâtel, Switzerland

## Abstract

Long-term social recognition is vital for species with complex social networks, where familiar individuals can encounter one another after long periods of separation. For non-human primates who live in dense forest environments, visual access to one another is often limited, and recognition of social partners over distances largely depends on vocal communication. Vocal recognition after years of separation has never been reported in any great ape species, despite their complex societies and advanced social intelligence. Here we show that bonobos, *Pan paniscus,* demonstrate reliable vocal recognition of social partners, even if they have been separated for five years. We experimentally tested bonobos’ responses to the calls of previous group members that had been transferred between captive groups. Despite long separations, subjects responded more intensely to familiar voices than to calls from unknown individuals - the first experimental evidence that bonobos can identify individuals utilising vocalisations even years after their last encounter. Our study also suggests that bonobos may cease to discriminate between familiar and unfamiliar individuals after a period of eight years, indicating that voice representations or interest could be limited in time in this species.

The social life of many primate species is characterised by lasting associations between individuals, making individualised social knowledge of primary importance. Individual vocal recognition has been found in many primate species^1^,^2^,^3^,^4^,^5^,^6^ and is particularly important for those living in dense forest habitats where vocalisations are often the most efficient communication channel^7^. For primate species with fission-fusion dynamics, community members regularly separate into smaller, fluid parties for hours, days or weeks while often maintaining vocal contact^8^. In many primate species, individuals disperse from their natal community during puberty^9^, but often continue to interact with former group members during subsequent inter-community encounters. Thus, successful social navigation within a community and between communities may depend on the ability to recognise both current and previous social partners. As a whole, long-term vocal recognition has only been demonstrated in a limited number of birds and mammals^10^,^11^,^12^,^13^,^14^, including only two monkey species with stable, non-fission-fusion group structures^15^,^16^ and has never been investigated in any ape species.

The present study focuses on the bonobo, an ape living in dense equatorial rainforest with large, overlapping home ranges and complex fission-fusion social networks between related and unrelated individuals^17^. It has been suggested that bonobos use vocalisations to communicate with distant conspecifics^7^,^8^, and some ape species have recently demonstrated a capacity for long-term memory for finding tools in the distant past, suggesting they could also retain social information for similar time periods^18^. We therefore hypothesised that long-term vocal recognition would be present in this species as a valuable adaptation for mediating their social environment. We used a series of playback experiments to test long-term vocal recognition in bonobos by comparing their behavioural responses to the vocalisations of familiar and unfamiliar conspecifics. Bonobos are known to display less aggression towards new individuals than their closest relatives, chimpanzees^19^,^20^. However, despite their relatively tolerant nature they still can react with mild aggression (with motor and vocal displays), caution or complete avoidance during intercommunity encounters^19^,^20^. This suggested that bonobos would likely react more cautiously (e.g. fewer approaches to the loudspeaker, less overall movement) to an unfamiliar voice than a familiar one, but that it would fail to induce extreme reactions such as aggression or panic. All familiar individuals had been separated from our tested subjects for varying numbers of years; in captivity individuals are sometimes transferred between zoos for population management and breeding programs. This, along with detailed life histories of individuals housed at three European zoos (Apenheul, Netherlands; Planckendael, Belgium, and La Vallée des Singes, France), allowed us to identify 15 individuals who had previously been housed with another individual living at one of the other zoos (Methods).

At each zoo, prior to the start of the playback experiment, we carefully mimicked a transfer of new bonobos and hid a loudspeaker in the enclosure where new individuals are normally held upon arrival. All of the subjects had previously experienced a real transfer event, by being transferred themselves (14 of 15 individuals), being in a group when a new individual was brought in (14 of 15) or both (13 of 15); therefore, this method increased the chances that they believed the broadcast calls were emitted by real individuals. At each zoo the experiment consisted of one mock transfer event followed by five playback trials, which occurred over a single day. After a playback trial, we waited until the whole group returned to ‘baseline’ behaviours, such as feeding, foraging, grooming or resting, before beginning the next trial (with a minimum of 10 minutes between trials). In keeping with the illusion that a real transfer was occurring, calls used for the playback stimuli were selected on the basis of acoustic similarities to vocalisations recorded during an actual transfer event (Fig. 1, Methods, Supplemental Table S1), and for each trial, the playback stimulus was composed of a unique call sequence (Fig. 1, Methods, Supplemental Audio 1). By using multiple observers we were able to test multiple subjects with each playback trial, this allowed us to minimise the number of playback trials in order to reduce the risk of habituation. Each observer recorded the behaviour of a single subject with a video camera. Each subject was recorded once in the familiar condition and once in the unfamiliar condition (See Methods).

## Results

### Vocal recognition of previous group members

**Figure 2:**
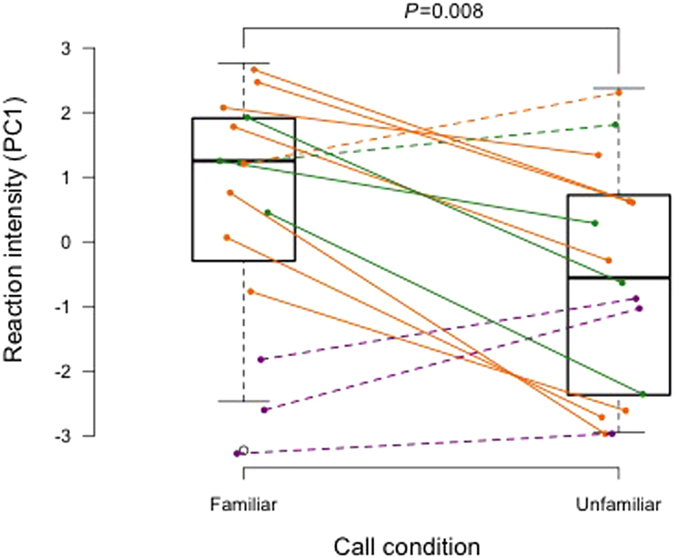
Bonobo reactions to the calls of familiar and unfamiliar individuals. Each individual was tested in both conditions, and each line on the figure links the responses in each condition for the same individual. The colour of the lines corresponds to the separation time between the subject and the former group mate used in the familiar condition. Green lines = bonobos that have been separated for 2–3 years (*n* = 4); orange lines = separated for 4.5–5.5 years (*n* = 8); purple lines = separated for 8–9 years (*n* = 3). The principle component score (PC1) represents an integrated measure of the behavioural response, with higher scores indicating a stronger behavioural reaction to the broadcast calls. Solid lines = bonobos that reacted more to the familiar voice; dashed lines = bonobos that reacted equally to both signals or more to the unfamiliar voice.

The bonobos’ reactions to the playbacks were assessed using 8 behavioural variables encompassing locomotion, looking direction and latency of behaviours after the playback (Methods). These measurements were then collapsed into a single composite behavioural score using a principle component analysis (Methods-Table 1). We found that bonobos responded more intensely when hearing a familiar voice compared to an unfamiliar voice (*n* = 15, linear mixed model, *t* = −0.396, *P* = 0.014, Methods-Table 2a). When hearing a familiar voice they responded more rapidly, increased their locomotion and approached the speaker more (Fig. 2, Supplemental Video 1). We also tested for any effects of the subject’s (the receiver) sex, rank and trial number. Additionally, as some individuals were subadults when housed with their previous social partner, any effect of age was tested (current age was used). Importantly, none of these factors were found to significantly influence the bonobos’ responses, highlighting the robustness of our findings.

### The effect of separation time on recognition

**Figure 3:**
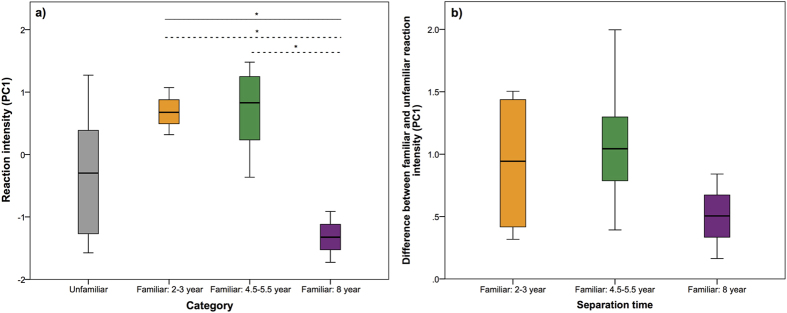
Effect of separation time on recognition. (**a)** Model 1 investigated the effects of the time of separation on the responses of subjects to a previous group member by comparing the reaction intensity (measured by the first principle component–PC1) between the three different separation categories (separated by 2–3 years, 4.5–5.5 years, or 8 years) (*p < 0.001; dashed lines are results of post-hoc comparisons). The PC1 scores for the unfamiliar condition are also presented on the graph as a reference. (**b)** Model 2 also investigated the effects of separation time by using a more conservative model comparing the absolute difference between the PC1 score for the unfamiliar and familiar conditions between the three separation categories. Despite showing the same pattern as model 1, the result of model 2 was not significant.

The playback experiments also allowed us to investigate the dynamics of long-term vocal recognition as familiar pairs (familiar condition) had been separated for varying numbers of years (separation time: 2–3 years, *n* = 4; 4.5–5.5 years, *n* = 8; 8–9 years, *n* = 3). Two statistical models were run to test the effect of separation time on the bonobos behavioural responses to familiar individuals. The first model investigated the magnitude of variation between the behavioural responses to each of the three separation time categories and found a significantly decreased response towards a past partner’s voice in dyads that had been separated for more than 8 years (*n* = 15; LMM*: t* = −5.230, *P* < 0.0001, Methods-Table 2b) (Fig. 3a). Post-hoc tests showed no significant differences in the bonobos’ reactions for pairs separated between 2–3 years and 4.5–5.5 years (multiple comparison test: *z* = −0.651, *P* = 0.784). Conversely, both were significantly different from the reactions of dyads separated for 8–9 years (multiple comparison tests between 2–3 years and 8–9 years: *z* = −4.802, *P* < 0.0001, between 4.5–5.5 years and 8–9 years: *z* = −6.707, *P* < 0.0001). As this first model only considered variation in the responses to familiar individuals, we analysed the data with a second model by measuring the absolute difference between each subjects’ response to familiar and unfamiliar individuals. While this second model shows the same trend as the first model (Fig. 3b), the difference between the three separation time groups was not significant (*n* = 15; LMM, *t* = −1.240, *P* = 0.226, Methods - Table 2c, Fig. 3b).

## Discussion

Here we provide the first experimental evidence that an ape species is capable of long-term vocal recognition of former social partners even after five years of separation. Our results demonstrate the importance of individualised vocal signalling for bonobos, which helps them navigate a complex fission-fusion society by maintaining communication between community members with whom they are out of physical range. In the current experiment we utilised more short-range calls than are typically displayed in these wild inter- and intra-community interactions^7^. However, within the bonobo vocal repertoire many call types are known to carry a variety of information–for instance, call sequences can carry information about different food types^21^ and copulation calls show individual vocal signatures^22^. Our results thus further demonstrate that loud long-calls are not alone in transmitting identity information, and that bonobos are able to use more short-ranged vocalisations to recognise familiar individuals. Additionally, in bonobos, inter-community encounters regularly begin with vocal exchanges, which appear to set the tone for the following interaction and generally result in either avoidance or peaceful interactions in which the two groups may even feed together^19^,^20^. In such a scenario, long-term vocal recognition enables bonobos to identify individuals without visual access, allowing them to favour meetings with affiliative individuals and avoid individuals with whom they have a conflictive relationship.

Despite the evidence of vocal recognition displayed by the experiment, our results suggest decreased reactions to former group members after eight years of separation, supported by statistical model 1 and underlying a possible limit to long-term vocal recognition (Fig. 3a). It is possible that bonobos are unable to recognise past social partners after a long period without contact, either because they cannot retain the memory of individual vocal signatures for longer than 6 to 8 years without reinforcement, or because a bonobo’s voice significantly changes over time as they age. As bonobos are very visual creatures, it could also be that long-term social recognition is strongest in the visual modality. Even a high degree of relatedness between the pairs in the eight-year separation category did not appear to secure long-term recognition (See Supplemental Table S2), despite the fact that mother-son bonds are particularly strong in bonobos^19^.

Alternatively, bonobos may in fact recognize the identity of the caller but are not motivated to react, as their social networks are highly dynamic, bonding and dominance between individuals may need to be reset after a significant period of separation.

However, we obtained contrasting support from the two statistical models testing the effects of long-term recognition. Despite a clear trend in the raw data (Fig. 3b), the more conservative model 2 did not support an upper limit of vocal recognition. Indeed, as bonobos have demonstrated advanced cognitive abilities and social skills^17^,^23^,^24^ and have performed well in memory tasks^25^, a social memory longer than 5 years was hypothesised. While long-term vocal memory has not been tested in any ape species or humans, both have demonstrated at least a decade long memory for the faces of former group mates, suggesting the potential for long-term social memory in the vocal modality^26^,^27^. A recent study showed that dolphins, another species with highly developed social and cognitive skills that also live in fission-fusion societies, could retain a vocal memory of conspecifics for decades^14^ and anecdotal evidence from another study suggests a similar vocal memory in African elephants^12^. Importantly, as the bonobos’ reactions were highly idiosyncratic we cannot exclude the possibility that this result is due to a low sample size (*n* = 3) for individuals separated for more than 8 years. This discrepancy between both models may be explained by our sample size; therefore, to conclusively demonstrate the upper limit of bonobo vocal recognition further investigation is required.

## Methods

### Subjects

In order to test the long-term vocal recognition of bonobos we benefited from the transfers of captive individuals between zoos for population management and breeding programs–these movements can, in a way, mimic their migration patterns in the wild. After examining the life histories of the 34 individuals housed in multi-female, multi-male groups between three European zoos (Apenheul, Netherlands; Planckendael, Belgium; La Vallée des Singes, France), we found 15 individuals who had been housed together in the past (either at one of the three zoos or at other zoos throughout Europe). We only included individuals who had been housed together over the age of seven. At the time of the experiment the youngest tested subject was 10 years old and the oldest 45 (mean age = 21; median age = 19).

Each pair had been housed together for long periods of time (from 4 to 17 years) but had since been housed separately for 2 to 9 years. The 15 subjects, aged 10 to 45, were equally balanced across sex (male *n* = 7; female *n* = 8), rank (High rank *n* = 5; mid-rank *n* = 6; low rank *n* = 4) and zoo (Apenheul *n* = 5; Planckendael *n* = 6; La Vallée des Singes *n* = 4). All individuals were subadults or adults (≥7 years-old) when housed with their previous group member and aged 10 or more during the experiment (Supplemental Table S2). At each zoo, an individual’s rank was assigned categorically based on the following: during agonistic encounters, whether mild such as food competition or severe such as conflicts resulting in serious injury, if an individual was most likely to be the aggressor they were classified as high-ranking, if an individual could be an aggressor or a victim, depending on the identity of the social partner during an event, they were classified as mid-ranking, and if an individual was most likely to the victim they were classified as low-ranking. Each rank assignment was then discussed and verified with a minimum of two keepers at each zoo.

### Playback Stimuli

Calls used for the playback stimuli were taken from a databank of vocal recordings from all individuals at the three zoos amassed by SK in 2013. Calls were selected on the basis of an acoustic similarity to vocalisations recorded during an actual transfer event–where two individuals were transferred together to La Vallée des Singes in 2012–and can be described as peep-yelps^28^ (See Supplemental Table S1 for acoustic details). Individual call sequences contained 4–6 calls and had decreasing intercall intervals along the progression of the sequence, as in the call sequence heard during the real transfer, with a mean total sequence time of 5.70 s (range of 3.79 s–7.882 s) for Apenheul and Planckendael. Because the enclosure at La Vallée des Singes is more than two-times larger than the enclosures at the other two zoos, the call sequences played there were followed by two additional calls 10 seconds later, to ensure all bonobos heard the playback sequences. All sequences were broadcast between 65–80 dB, SPL measured at 1 meter from the loudspeaker.

### Playback Experiments

At each zoo the experiment began with a mock transfer of new bonobos, following their standard transfer procedure, respectively. Everything was done as if real bonobos were being moved–a truck/tractor was driven up to the building carrying a crate, the crate was placed to the slide opening to the separation cage, and the slides were opened and closed. As all of the subjects had previously experienced a real transfer event in similar conditions, we expected that the mimicked transfer would have bonobos believe that other individuals were arriving. During this time a loudspeaker (Juster Elite Speaker for Apenheul and Planckendael, and Western Rivers Nite Stalker Pro for La Vallée des Singes) was placed in a separation enclosure where new bonobos are normally kept upon arrival. All physical and visual access to this enclosure was blocked during the experiment and for at least 12 hours before the mock transfer. Beginning from 10–15 minutes prior to the mock transfer three to four observers set-up around the cages where bonobos are usually housed, and where the experiment was to occur. The bonobos at all three zoos are regularly observed by researchers and did not show any visible signs of disturbance. At each zoo the experiment consisted of a single mock transfer followed by a total of five playback trials. After the mock transfer we waited until the group returned to baseline behaviours-resting, foraging or grooming-before broadcasting the first playback. Before proceeding from the first to subsequent playbacks we again waited until the group returned to baseline behaviours–therefore time between playbacks varied from 10 minutes to 37 minutes (mean = 25 minutes) for Apenheul and Planckendael. Due to environmental conditions at La Vallée des Singes the first playback occurred four hours before the proceeding four trials (which then averaged 44 minutes between broadcasts).

Each playback, whether familiar or unfamiliar, contained a unique, acoustically distinct, set of calls. For all three zoos the voice of each familiar individual was broadcast only once (number of past group members used for the familiar trials: Planckendael, *n* = 4; Apenheul, n = 3; La Vallée des Singes, *n* = 2). The call sequences used for the unfamiliar trials at Apenheul came from a single female unknown to all individuals in the group, this was also the case for La Vallée des Singes. At Planckendael, there was not a single individual within our database that was unknown to everyone; therefore at this zoo each broadcast individual was familiar to some and unfamiliar to others (see Supplementary Table S4).

In total each subject was tested once in each of the two experimental conditions. As the enclosures at each zoo were of different sizes, shapes and contained different climbing structures, a precise distance from the speaker across all subjects during playback trials could not be set. To control for this variation we able to test the majority of individuals at roughly the same distance from the speaker for both of their playback trials (Within 0–2 metres of same position for both trials, *n* = 12; within 2–4 metres, *n* = 2; > 4 metres, *n* = 1; See Supplemental Table S3). Additionally, the order in which each bonobo heard the stimulus for each condition was counterbalanced (8 individuals heard the familiar first while 7 heard the unfamiliar first).

Due to limitations in the number of researchers able to observe during the experiments we were not able to test all individuals in the first two playback trials. As such, some individuals were exposed to familiar and/or unfamiliar voices before their responses to either or both conditions could be recorded (See Supplemental Table S4). We controlled for stimuli exposure (we did not differentiate between familiar and unfamiliar playbacks) prior to their own trials by including trial number as a random factor in the model.

To be able to test all 15 subjects in a realistic situation, we found that not disturbing the groups’ normal composition was the best option. While this choice led to the potential for pseudoreplication, it avoided the stress caused to individuals by separating their normal group and allowed us to test multiple individuals at once, which helped to avoid habituation. This possible non-independence of the reactions of tested individuals, together with the fact that we took multiple individuals’ reactions from the same playback trial, was controlled for by entering trial number as a random factor in statistical models (see statistical analysis of behavioural reactions below).

### Measurements of behavioural responses

Each observer filmed (Handheld camera models: Canon Legria FS406 and Canon Legria HF200; Stable camera models : GoPro Hero3 and JVC GC-XA1 Adixxion HD) one focal subject that was randomly assigned. Subjects were video recorded for 10 minutes before and 10 minutes after each playback trial. SK coded all videos. To ensure unbiased coding, all videos were given numbers and coded blind to the condition a minimum four weeks after the experiment occurred. To inform which behavioural measures would be included we relied on reported behaviours when bonobos encounter neighbouring groups in the wild^20^, observations by SK during a transfer of a female into the Apenheul group and on previous studies investigating vocal recognition in a variety of species^2^,^4^,^11^,^16^,^29^. As stated in the introduction, bonobos are known for their relatively tolerant nature towards foreign individuals, therefore we expected a mild intensity reaction to the playback broadcasts. We assessed a variety of measures on body and head movements, in relation to the speaker and in general. Social interactions and vocal responses were also coded; however, our playbacks elicited no interactions between individuals (neither aggressive or affiliative) and only one vocalisation (a single call by an unidentified individual). Therefore, the following eight behavioural variables, measured in the 60 seconds following the playback, were included:

- latency after the start of the playback to the first behaviour displayed. It could have been any behaviour or the cessation of a behaviour–for instance if they were eating and stopped;

- latency to the first locomotion after the start of the playback broadcast;

- total duration of locomotion in any direction;

- total duration of locomotion toward the speaker;

- number of separate locomotion occurrences;

- duration of time spent looking toward the speaker;

- number of times an individual looked toward the speaker;

- total number of head movements (each change of head direction was counted as one movement event);

To conduct inter-observer reliability FL coded 67% of the videos as above and results were compared for each variable separately. The intraclass correlation coefficient (ICC) was above 0.860 for all variables (Latency to first behaviour = 0.868; latency to first locomotion = 0.908; duration of locomotion (in any direction) = 0.984; duration of locomotion toward speaker = 0.969; number of locomotion events = 0.878; duration looking toward speaker = 0.895; number of head movements oriented toward speaker = 0.920; total number of all head movements = 0.874).

Instead of separately analysing the 8 dependent behavioural measures, we performed a principal component analysis (PCA) and retained a single composite score. By using a PCA, we approached a Gaussian distribution, built an integrated measure of the behavioural response and demonstrated which behavioural variables were important. As shown in Table 1, latency to the first locomotion, duration of locomotion, the number of movements, and the propensity to move towards the loudspeaker were the main factors that loaded on the first PC score (PC1). PC1 was thus chosen as a unique composite score representing the strength of an individual’s behavioural response to a playback (with positive scores indicating a stronger behavioural response and negative scores representing a reduced reaction).

### Statistical analysis of behavioural reactions

Each individual (*n* = 15) was tested with one familiar voice and one unfamiliar voice. The eight behavioural variables were measured in the 60 seconds following the playback and used in a principle component analysis, resulting in one principle component (PC1), which explained 42.2% of the variation in the data. To test for an effect of familiarity versus non-familiarity of the playback stimuli on the bonobos’ behavioural response, we used a linear mixed effect model with PC1 as the dependent measure (R package lme4), after checking the distribution of the residuals with respect to normality and homoscedasticity (fixed effects: subject rank, subject sex, subject age; random effects: individual identity, playback trial number, zoo location). *P* values were obtained with likelihood-ratio tests comparing the fit of the full model with reduced models lacking fixed effects. To test for the effect of separation time, we used two different models. The first model was restricted to experiments with calls from past (familiar) partners. This analysis was followed by post-hoc multiple comparison tests (function glht in multcomp R package). Given individual differences in reactivity to playbacks and the small number of individuals having experienced the same separation time, we used a second, more conservative, model that took into account the relative difference in response to familiar and unfamiliar calls (for each individual we calculated the absolute difference in their PC1 score between the two conditions). The degree of relatedness between the pairs was initially considered, however it was not balanced across separation time conditions, and therefore could not be accurately tested (all three of the eight-year separation category were 1^st^ degree related, while only one of the other 12 pairs was–Supplemental Table S2).

### Ethics Statement

All work was performed in accordance with the relevant guidelines and regulations, and all experimental protocols were approved by the Institutional Animal Ethical Committee of the University of Lyon/Saint-Etienne, under the authorization no. 42-218-0901-38 SV 09 (Lab ENES).

## Additional Information

**How to cite this article**: Keenan, S. *et al.* Enduring voice recognition in bonobos. *Sci. Rep.* **6**, 22046; doi: 10.1038/srep22046 (2016).

## Supplementary Material

Supplementary Information

Supplementary Video 1

Supplementary Audio 1

## Figures and Tables

**Table 1:** Factor Loadings of measured behavioural variables on the first Principal Component (PC1).

Behavioural variables	Factor loading score
	PC1
	−0.176
**Latency to first locomotion**	**−0.778**
**Duration of locomotion (in any direction)**	**0.873**
**Duration of locomotion toward speaker**	**0.771**
**Number of locomotion events**	**0.791**
Duration looking toward speaker	0.488
Number head movements oriented toward speaker	0.573
Total number of all head movements	0.437

Factors that loaded highly onto PC1 are in bold.

**Table 2:** Results of LME models.

	Estimate	Standard Error	t	*P*
(a) MODEL 1				
(Intercept)	2.539	2.197	1.156	
Trial Condition (Familiar V. Unfamiliar)	−1.151	2.364	−0.396	0.014
Subject Rank	0.801	0.755	1.060	0.321
Subject Sex	−0.696	1.006	−0.692	0.510
Subject Age	0.012	0.057	0.302	0.852
(b) MODEL 2				
(Intercept)	2.491	1.713	1.454	
Separation Time	−0.066	0.013	−5.230	< 0.0001
Subject Rank	0.526	0.513	1.024	0.342
Subject Sex	0.625	0.713	0.876	0.388
Subject Age	0.030	0.041	0.734	0.516
(c) MODEL 3				
(Intercept)	1.737	0.625	2.781	
Separation Time	−0.088	0.071	−1.240	0.226
Subject Rank	−0.398	0.474	−0.840	0.394
Subject Sex	0.444	0.337	1.317	0.200
Subject Age	−0.02	0.022	−0.944	0.352

(**a**) Model 1 tested for the effect of vocal familiarity on bonobos’ response to playbacks. (**b,c**) Models 2 and 3 tested the effect of separation time on bonobos’ response to previous partner’s voice. Model 2 examines responses to the familiar individual playback alone while model 3 uses the relative difference for each individual in response to familiar vs. unfamiliar individuals. *n* = individuals.

**Figure 1:**
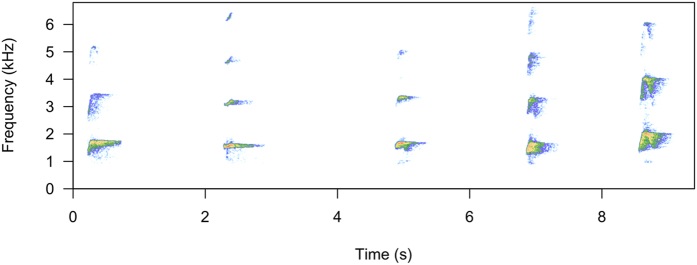
An example of a playback stimulus. This sequence was produced by an adult bonobo female living at Apenheul Zoo and was used to test a familiar condition at Planckendael Zoo.

## References

[b1] Cheney D. L. & Seyfarth R. M. Vocal recognition in free-ranging vervet monkeys. Animal Behaviour 28, 362–364 (1980).

[b2] Rendall D., Rodman P. S. & Emond R. E. Vocal recognition of individuals and kin in free-ranging rhesus monkeys. Animal Behaviour 51, 1007–1015 (1996).

[b3] Kojima S., Izumi A. & Ceugniet M. Identification of vocalizers by pant hoots, pant grunts and screams in a chimpanzee. Primates 44, 225–230 (2003). 12884113 10.1007/s10329-002-0014-8

[b4] Ramos-Fernández G. Vocal Communication in a Fission-Fusion Society: Do Spider Monkeys Stay in Touch With Close Associates? International Journal of Primatology, 26 1077–1092 (2005).

[b5] Miller C. T. & Thomas A. W. Individual recognition during bouts of antiphonal calling in common marmosets. Journal of Comparative Physiology 198, 337–346 (2012). 22277952 10.1007/s00359-012-0712-7 PMC3799814

[b6] Candiotti A., Zuberbuhler K. & Lemasson A. Voice discrimination in four primates. Behavioural Processes 99, 67–72 (2013). 23800631 10.1016/j.beproc.2013.06.010

[b7] Hohmann G. & Fruth B. Structure and use of distance calls in wild bonobos (*Pan paniscus*). International Journal of Primatology 15, 767–782 (1994).

[b8] White F. J. In Great Ape Societies (eds McGrew W. C., Marchant L. F. & Nishida T.) 29–41 (Cambridge Univ. Press, Cambridge, 1996).

[b9] Pusey A. E. & Packer C. In Primate Societies (eds Smuts B. B., Cheney D. L., Seyfarth R. M., Wrangham R. W. & Struhsaker T. T.) 250–266 (University of Chicago Press, Chicago, 1987).

[b10] Godard R. Long-term memory of individual neighbours in a migratory songbird. Nature 350, 228–229 (1991).

[b11] Insley S. J. Long-term vocal recognition in the northern fur seal. Nature 406, 404–405 (2000). 10935635 10.1038/35019064

[b12] McComb K., Moss C., Sayialel S. & Baker L. Unusually extensive networks of vocal recognition in African elephants. Animal Behaviour 59, 1103–1109 (2000). 10877888 10.1006/anbe.2000.1406

[b13] Boeckle M. & Bugnyar T. Long-term memory for affiliates in ravens. Current Biology 22, 801–806 (2012). 22521788 10.1016/j.cub.2012.03.023 PMC3348500

[b14] Bruck J. N. Decades-long social memory in bottlenose dolphins. Proceedings of the Royal Society B 280, 1–6 (2013). 10.1098/rspb.2013.1726 PMC3757989 23926160

[b15] Lemasson A. & Hausberger M. Acoustic variability and social significance of calls in female Campbell’s monkeys (*Cercopithecus campbelli campbelli*). The Journal of the Acoustical Society of America 129, 3341–3352 (2011). 21568434 10.1121/1.3569704

[b16] Matthews S. & Snowdon C. T. Long-term memory for calls of relatives in cotton-top tamarins (*Saguinus oedipus*). Journal of Comparative Psychology 125, 366–369 (2011). 21574684 10.1037/a0023149 PMC3191862

[b17] Kanō T. The last ape: Pygmy chimpanzee behavior and ecology. (Stanford University Press, Stanford, California, 1992).

[b18] Martin-Ordas G., Berntsen D. & Call J. Memory for distant past events in chimpanzees and orangutans. Current Biology 23, 1438–1441 (2013). 23871242 10.1016/j.cub.2013.06.017

[b19] Furuchi T. Female contributions to the peaceful nature of bonobo society. Evolutionary Anthrophology 20, 131–142 (2011). 10.1002/evan.20308 22038769

[b20] Hohmann G. & Fruth B. In Behavioural Diversity in Chimpanzees and Bonobos (eds Boesch C., Hohmann G. & Marchant L. F.) 138–150 (Cambridge Univ. Press, Cambridge, 2002).

[b21] Clay Z. & Zuberbuhler K. Bonobos extract meaning from call sequences. PloS One 6, e18786 (2011). 21556149 10.1371/journal.pone.0018786 PMC3083404

[b22] Clay Z. & Zuberbuhler K. The Structure of Bonobo Copulation Calls During Reproductive and Non-Reproductive Sex. Ethology 117, 1158–1169 (2011).

[b23] Hare B., Melis A. P., Woods V., Hastings S. & Wrangham R. Tolerance allows bonobos to outperform chimpanzees on a cooperative task. Current Biology 17, 619–623 (2007). 17346970 10.1016/j.cub.2007.02.040

[b24] Herrmann E, Hare B, Call J & Tomasello M. Differences in the Cognitive Skills of Bonobos and Chimpanzees. PLoS One 5, e12438 (2010). 20806062 10.1371/journal.pone.0012438 PMC2929188

[b25] Martin-Ordas G., Haun D., Colmenares F. & Call J. Keeping track of time: evidence for episodic-like memory in great apes. Animal Cognition 13, 331–340 (2010). 19784852 10.1007/s10071-009-0282-4 PMC2822233

[b26] Bahrick H. P., Bahrick P. O. & Wittlinger R. P. Fifty years of memory for names and faces: a cross sectional approach. Journal of Experimental Psychology: General 104, 54–75 (1975).

[b27] Hanazuka Y., Shimahara N., Tokuda Y. & Midorikawa A. Orangutans (Pongo pygmaeus) remember old acquaintances. PLoS One 8, e82073 (2013). 24324746 10.1371/journal.pone.0082073 PMC3852890

[b28] deWaal F. B. M. The communicative repertoire of captive bonobos (Pan paniscus) compared to that of chimpanzees. Behaviour 27, 183–251 (1988).

[b29] Bergman T. J. Experimental evidence for limited vocal recognition in a wild primate: implications for the social complexity hypothesis. Proceedings of the Royal Society B 277, 3045–3053 (2010). 20462901 10.1098/rspb.2010.0580 PMC2982026

