# Supplementary material for: Enduring voice recognition in bonobos
Source: Sci Rep. 2016 Feb 25;6:22046. doi: 10.1038/srep22046 (PMC4766561; doi:10.1038/srep22046)
Supplement: Supplementary Information [file srep22046-s1.doc]

**Supplemental Information**

*for*

**Enduring voice recognition in bonobos**

Sumir Keenan^1,2^, Nicolas Mathevon^1,3^, Jeroen MG Stevens^4^, Jean Pascal Guéry^5^, Klaus Zuberbühler^2,6^ and Florence Levréro^1^

^1^Université de Lyon/Saint-Etienne, Equipe Neuro-Ethologie Sensorielle, ENES/Neuro-PSI, CNRS UMR 9197, Saint-Etienne, France

^2^University of St. Andrews, Department of Psychology & Neuroscience, St. Andrews, Scotland, UK

^3^Department of Psychology, Hunter College, CUNY, New York, USA

^4^Royal Zoological Society of Antwerp, Centre for Research and Conservation, Antwerp, Belgium

^5^Vallée des Singes Zoological Park, Romagne, France

^6^Université de Neuchâtel, Department of Comparative Cognition, Neuchâtel, Switzerland

**Supplemental Table S1.** Acoustic features of the calls recorded during a real transfer event and the calls used for the broadcast sequences in the playback experiments.

|  |  | Calls from real transfer event (*n*= 8) | Calls used for playbacks (*n*=50) |
| --- | --- | --- | --- |
| Start Frequency  (Hz) | Mean | 1683 | 1542 |
|  | S.D. | 120 | 218 |
|  | Min | 1497 | 1138 |
|  | Max | 1821 | 2309 |
| End Frequency (Hz) | Mean | 1772 | 1580 |
|  | S.D. | 133 | 209 |
|  | Min | 1596 | 1227 |
|  | Max | 1896 | 2021 |
| Frequency modulation (Hz) | Mean | 90 | 37 |
|  | S.D. | 47 | 129 |
|  | Min | 0 | -288 |
|  | Max | 150 | 316 |
| Duration (s) | Mean | .231 | .224 |
|  | S.D. | .070 | .075 |
|  | Min | .12 | .14 |
|  | Max | .32 | .51 |

**Supplemental Table S2**. Tested subjects (age, sex, zoo of residence, rank) and their relationship with the individuals whose voices were broadcast in the familiar condition during playback experiments.

| Subject | Age | Sex | Residence | Rank | Familiar Call Conditions | | | |
| --- | --- | --- | --- | --- | --- | --- | --- | --- |
|  |  |  |  |  | Separation Time (months) | Name of familiar | Sex of familiar | Subject and familiar relation |
| Daniela | 45 | F | Vallee des Singes | High | 54 | Lina | F | Unrelated |
| Kirembo | 21 | M | Vallee des Singes | Low | 54 | Lina | F | Unrelated |
| Diwani | 17 | M | Vallee des Singes | Low | 54 | Louisoko | M | Paternal half-brother |
| David | 12 | M | Vallee des Singes | Mid | 54 | Louisoko | M | Unrelated |
| Lina | 28 | F | Planckendael | High | 54 | Khaya | F | Unrelated |
| Louisoko | 16 | M | Planckendael | Mid | 54 | Khaya | F | Unrelated |
| Lucuma | 11 | M | Planckendael | Low | 54 | Khaya | F | Unrelated |
| Djanoa | 19 | F | Planckendael | Mid | 65 | Hortense | F | Unrelated |
| Vifijo | 19 | M | Planckendael | Low | 100 | Hortense | F | Mother-son |
| Busira | 10 | F | Planckendael | Low | 32 | Lisala | F | Unrelated |
| Jill | 29 | F | Apenheul | High | 33 | Lingala | F | Mother-daughter |
| Hortense | 36 | F | Apenheul | Mid | 100 | Vifijo | M | Mother-son |
| Zuani | 22 | F | Apenheul | High | 33 | Lingala | F | Unrelated |
| Kumbuka | 14 | F | Apenheul | Mid | 33 | Lingala | F | Unrelated |
| Zamba | 16 | M | Apenheul | Mid | 100 | Vifijo | M | Full Brothers |

**Supplemental Table S3.** Playback experimental details for each subject’s familiar and unfamiliar conditions, including the principal component scores (PC1) calculated from their behavioural reactions to the playback stimuli.

|  |  | Familiar Condition | | | | Unfamiliar Condition | | | |
| --- | --- | --- | --- | --- | --- | --- | --- | --- | --- |
| Subject | Zoo | Play-back Trial No. | Distance from speaker (in metres) | Individuals familiar to PB voice **/** Total number of individuals in group* | PC1 Score | Play-back Trial No. | Distance from speaker  (in metres) | Individuals familiar to PB voice **/** Total number of individuals in group* | PC1  Score |
| Hortense | Apenheul** | 3 | 6 | 0/5 | -1.727 | 1 | 6 | 0/5 | -1.563 |
| Jill | Apenheul** | 2 | 8 | 3/5 | 0.683 | 1 | 8 | 0/5 | 1.001 |
| Kumbuka | Apenheul** | 2 | 4 | 1/2 | 1.072 | 4 | 2 | 0/2 | -0.298 |
| Zamba | Apenheul** | 3 | 2 | 0/2 | -1.324 | 1 | 2 | 0/2 | -0.483 |
| Zuani | Apenheul** | 2 | 5 | 3/5 | 0.317 | 3 | 6 | 0/5 | -1.187 |
| Busira | Planck | 3 | 8 | 0/8 | 0.669 | 1 | 8 | 3/8 | 0.153 |
| Djanoa | Planck | 1 | 5 | 2/8 | 0.974 | 2 | 12 | 4/8 | -0.134 |
| Lina | Planck | 4 | 8 | 3/8 | -0.363 | 3 | 8 | 1/8 | -1.350 |
| Louisoko | Planck | 4 | 12 | 3/8 | 1.479 | 3 | 8 | 1/8 | 0.390 |
| Lucuma | Planck | 4 | 8 | 3/8 | 1.381 | 3 | 8 | 1/8 | 0.383 |
| Vifijo | Planck | 1 | 4 | 2/8 | -0.912 | 2 | 3 | 4/8 | -0.407 |
| Daniela | VDS | 1 | 7 | 4/14 | 1.116 | 2 | 7 | 0/14 | 0.723 |
| David | VDS | 3 | 9 | 4/14 | 0.046 | 4 | 9 | 0/14 | -1.442 |
| Diwani | VDS | 3 | 14 | 4/14 | 0.422 | 4 | 18 | 0/14 | -1.575 |
| Kirembo | VDS | 1 | 9 | 4/14 | 0.684 | 2 | 9 | 0/14 | 1.271 |

*Number of individuals in each group familiar to the voice in the playback broadcast, excluding subject **/** total number of individuals in the group, including subjects and non-subjects but excluding individuals under the age of four. **The Apenheul group have lived in two sub-groups since 2013, during the playback experiment Zamba and Kumbuka were completely separate from the other individuals - therefore, total number of familiar individuals listed in this column only includes individuals within his or her sub-group and not across the whole Apenheul population.

**Supplemental Table S4:** Details on each playback trial at each zoo, including when each individual was recorded for the familiar and unfamiliar conditions and their familiarity to each playback.

**a) Apenheul**

| **Playback Trial Number** | **Playback Stimulus** | **Hortense** | **Jill** | **Kumbuka** | **Zamba** | **Zuani** |
| --- | --- | --- | --- | --- | --- | --- |
| 1 | Lisala #1 | **Observed - Unfamiliar** | **Observed - Unfamiliar** | Present - Unfamiliar | **Observed - Unfamiliar** | Present - Unfamiliar |
| 2 | Lingala #1 | Present - Familiar | **Observed - Familiar** | **Observed - Familiar** | Present - Familiar | **Observed - Familiar** |
| 3 | Vifijo #1 | **Observed - Familiar** | Present - Unfamiliar | Present - Unfamiliar | **Observed - Familiar** | **Observed - Unfamiliar** |
| 4 | Lisala #2 | Present - Unfamiliar | Present - Unfamiliar | **Observed - Unfamiliar** | Present - Unfamiliar | Present - Unfamiliar |

**b) Planckendael**

| **Playback Trial Number** | **Playback Stimulus** | **Busira** | **Djanoa** | **Lina** | **Louisoko** | **Lucuma** | **Vifijo** |
| --- | --- | --- | --- | --- | --- | --- | --- |
| 1 | Hortense #1 | **Observed - Unfamiliar** | **Observed - Familiar** | Present - Unfamiliar | Present - Unfamiliar | Present - Unfamiliar | **Observed - Familiar** |
| 2 | Daniela #1 | Present - Unfamiliar | **Observed - Unfamiliar** | Present - Familiar | Present - Familiar | Present - Familiar | **Observed - Unfamiliar** |
| 3 | Lisala #1 | **Observed - Familiar** | Present - Unfamiliar | **Observed - Unfamiliar** | **Observed - Unfamiliar** | **Observed - Unfamiliar** | Present - Unfamiliar |
| 4 | Khaya #1 | Present - Unfamiliar | Present - Unfamiliar | **Observed - Familiar** | **Observed - Familiar** | **Observed - Familiar** | Present - Unfamiliar |

**c) La Vallée des Singes**

| **Playback Trial Number** | **Playback Stimulus** | **Daniela** | **David** | **Diwani** | **Kirembo** |
| --- | --- | --- | --- | --- | --- |
| 1 | Lina #1 | **Observed - Familiar** | Present - Familiar | Present - Familiar | **Observed - Familiar** |
| 2 | Djanoa #1 | **Observed - Unfamiliar** | Present - Unfamiliar | Present - Unfamiliar | **Observed - Unfamiliar** |
| 3 | Louisoko #1 | Present - Familiar | **Observed - Familiar** | **Observed - Familiar** | Present - Familiar |
| 4 | Djanoa #2 | Present - Unfamiliar | **Observed - Unfamiliar** | **Observed - Unfamiliar** | Present - Unfamiliar |

Trials marked as **‘Observed’** are each individuals trials that were included in the analysis for the current study, along with the trial’s condition. Five playbacks occurred at each zoo, however only the first four were retained for analysis.

**Supplementary Audio 1**: (File name: Supplementary Audio 1.wav) Example of a playback stimulus used in the familiar condition.

**Supplementary Video 1**: (File name: Supplementary Video 1.mp4) Example of a bonobo’s behavioural response to the voice of a past social partner (familiar condition).
